# Long-term performance of an external stent for saphenous vein grafts: the VEST IV trial

**DOI:** 10.1186/s13019-018-0803-9

**Published:** 2018-11-19

**Authors:** David P. Taggart, Carolyn M. Webb, Anthony Desouza, Rashmi Yadav, Keith M. Channon, Fabio De Robertis, Carlo Di Mario

**Affiliations:** 1 Nuffield Department of Surgery, University of Oxford, John Radcliffe Hospital, Oxford, UK; 2 National Heart & Lung Institute, Imperial College London, London, UK; 3 Department of Cardiology, Royal Brompton Hospital, Sydney Street, London, SW3 6NP UK; 4 Department of Cardiothoracic Surgery, Royal Brompton Hospital, Sydney Street, London, UK; 5 Department of Cardiovascular Medicine, University of Oxford, John Radcliffe Hospital, Oxford, UK; 6 Department of Cardiothoracic Surgery, Harefield Hospital, Middlesex, London, UK

**Keywords:** Coronary artery bypass graft surgery, Saphenous vein graft, Intimal hyperplasia, External stent

## Abstract

**Background:**

Externally stenting saphenous vein grafts reduces intimal hyperplasia, improves lumen uniformity and reduces oscillatory shear stress 1 year following surgery. The present study is the first to present the longer-term (4.5 years) performance and biomechanical effects of externally stented saphenous vein grafts.

**Methods:**

Thirty patients previously implanted with the VEST external stent in the randomized, within-patient-controlled VEST I study were followed up for adverse events; 21 of these were available to undergo coronary angiography and intravascular ultrasound.

**Results:**

Twenty-one stented and 29 nonstented saphenous vein grafts were evaluated by angiography and ultrasound at 4.5 ± 0.3 years. Vein graft failure rates were comparable between stented and nonstented grafts (30 and 23% respectively; *p* = 0.42). All failures were apparent at 1 year except for one additional nonstented failure at 4.5 years. In patent vein grafts, Fitzgibbon perfect patency remained significantly higher in the stented versus nonstented vein grafts (81 and 48% respectively, *p* = 0.002), while intimal hyperplasia area (4.27 mm^2^ ± 1.27 mm^2^ and 5.23 mm^2^ ± 1.83 mm^2^ respectively, *p* < 0.001) and thickness (0.36 mm ± 0.09 mm and 0.42 mm ± 0.11 mm respectively, *p* < 0.001) were significantly reduced. Intimal hyperplasia proliferation correlated with lumen uniformity and with the distance between the stent and the lumen (*p* = 0.04 and *p* < 0.001 respectively).

**Conclusions:**

External stenting mitigates saphenous vein graft remodeling and significantly reduces diffuse intimal hyperplasia and the development of lumen irregularities 4.5 years after coronary artery bypass surgery. Close conformity of the stent to the vessel wall appears to be an important factor.

**Trial registration:**

NCT01415245. Registered 11 August 2011.

## Background

Saphenous vein grafts (SVG) remain the most commonly used conduit in coronary artery bypass grafting (CABG). However, despite significant advances in understanding SVG pathophysiology post implantation, relatively little progress has been made in the clinical setting - current vein graft failure rates range from 35 to 50% 5 to 10 years following CABG surgery [1]. SVG failure is attributed to the onset of structural changes in the conduit immediately post implantation due to its exposure to the hemodynamics of the arterial circulation [2]. SVG remodeling includes an inflammatory response accompanied by the development of intimal hyperplasia (IH) that serves as the foundation for graft thrombosis and occlusive atherosclerosis [2, 3]. Attempts to mitigate IH in vein grafts have been the focus of intense clinical research. However, most of the pharmacological therapeutic modalities have had a limited effect in the clinical setting, except for statins and beta-blockers [4–6]**.**

External stenting of SVGs has long been postulated to have the potential to lessen adverse mechanical and structural changes that contribute to SVG deterioration. In 2015 we reported our first in human experience with a new cobalt chrome external stent for SVGs (VEST, Vascular Graft Solutions, Israel) [7]. At 1 year, externally stented SVGs showed significant reduction in IH, improved lumen uniformity and reduced oscillatory shear stress compared to non-stented SVGs [7]. Conversely, a notable limitation was the lower patency rate of externally stented SVGs to the right compared to the left coronary vessels at 1 year [7]. As recently reported in the VEST II trial in this journal, avoidance of both metal clip ligation of SVG side branches and of fixation of the stent to the anastomoses significantly improved early patency rates of SVGs to the right coronary territory from 55 to 86% [8]. Currently two larger trials of external stenting are underway. VEST III (NCT02511834) is a European multicentre study that completed recruitment of 180 patients in January 2017. The study endpoints are major cardiovascular and cerebral events (MACCE) and graft patency as assessed by CT angiography at 6 months and 2 years. In the USA a similar 224 patient trial has recently started recruiting under the auspices of the Cardiothoracic Surgical Trials Network (VEST Pivotal, NCT 03209609). In the meantime, while several groups have reported 1-year patency rates there are no data in the literature regarding patency rates beyond this. The objective of the current VEST IV study was to investigate the effects of mechanical external stents 4–5 years after CABG.

## Methods

### Patients and design

The VEST I study prospectively enrolled 30 CABG patients with multi-vessel disease who were implanted with the VEST external stent (Vascular Graft Solutions, Tel Aviv, Israel) in a within-patient randomized controlled design, from October 2011 to September 2012 [7]. Eligible patients were scheduled for on-pump multi-vessel CABG including a left internal mammary artery to the left anterior descending coronary artery and SVGs to right and circumflex territories. Eligibility required a target vessel diameter ≥ 1.5 mm with a coronary stenosis > 75% and with an adequate distal vascular bed, as assessed by preoperative angiography. Each patient received one external stent device to a single SVG, randomly assigned intra-operatively by the opening of a sealed envelope only after all distal anastomoses were performed, to either the right or the circumflex coronary territories. One or more SVG remained non-stented and served as the control group. All SVGs were harvested in an open technique. Transit Time Flow Measurement (TTFM) was determined in all grafts. There was no significant difference in baseline grafting variables between stented and control groups (Table 2 of reference [7]). The primary effectiveness endpoint compared intimal hyperplasia area between groups, assessed by intravascular ultrasound (IVUS) at 12 months.

In the current extended follow-up study (VEST IV), MACCE data were collected for all patients. MACCE was defined as the composite occurrence of all-cause mortality, myocardial infarction (MI), revascularization, and/or stroke. Only patients who were alive and last known to have at least one patent vein graft were invited to return for a coronary angiogram and IVUS. Patients who did not have angiography underwent telephone follow-up. The study protocol conformed to the Declaration of Helsinki and was approved by a UK Research Ethics Committee. Each patient gave their informed consent to participate in the study.

### Angiography and IVUS

Contrast angiography was attempted for all grafts using the same camera angles as in VEST I, and using an identical method of analysis by the same core laboratory. Quantitative coronary angiography (QAngio XA®, Medis, The Netherlands) was performed for all patent grafts. Fitzgibbon grade I, II, or III was assigned to each graft to determine lumen uniformity: I, no intimal irregularity; II/III, irregularity of the intimal surface of < 50 and > 50% respectively [9].

IVUS examination was performed along the entire length of patent vein grafts (40 MHz IVUS catheter with automated 1.0 mm/s pullback, Boston Scientific, Hemel Hempstead, UK). Individual cross sectional IVUS images, approximately 10 mm apart over the entire vein graft length, were analyzed using QIVUS software (QIVUS® Medis, The Netherlands). Lumen and external elastic membrane (EEM) were identified and marked in accordance with convention [10] by an independent observer. IH area was calculated for every cross-section as the area between the EEM and the lumen perimeters. The IH thickness was calculated as the mean EEM radius minus the mean lumen radius. The VEST was identified and marked in the stented grafts and its distance from the lumen was calculated by subtracting the mean lumen radius from the mean VEST radius. To assess IVUS intra-observer variability, 11 pullbacks from 6 vein grafts (3 stented and 3 nonstented) were measured 1 month apart. IVUS intra-observer variability for EEM cross sectional area was − 0.02 +/− 0.6mm^2^ (*r* = 0.99, *p* = 0.99), and for lumen was 0.03 +/− 0.24 mm^2^ (*r* = 0.99, *p* = 0.97).

The primary efficacy endpoint compared IH area and thickness between stented and nonstented groups. Graft occlusion and Fitzgibbon perfect patency rates were also compared between the groups. The interaction between various disease markers obtained from the two imaging modalities was quantified by comparing the cross-sectional parameters derived from IVUS across angiographically perfectly patent (Fitzgibbon I) and patent with irregularities (Fitzgibbon II/III) graft groups. The effect of external stent dimensional conformity to the vessel wall was assessed by computing the correlation between stent distance from the lumen and IH thickness for the stented grafts.

### Statistical analysis

Descriptive statistics are provided as mean, standard deviation, minimum, median and maximum for continuous variables and as count and percent for non-continuous variables. Statistical testing was done using a mixed-effect model repeated measures, using measures taken at 1 and 4.5 year follow-ups. Continuous variables were analyzed using a linear regression model with ordinal variables analyzed using logistic regression. In both cases, subject was included as a random variable in the model. *P*-values of the device effect were obtained based on least squares mean estimates over both time points, since no interaction with time was obtained. Missing values at 4.5 years were inputed by using last observation carried forward from 1 year and included in the model. Pearson correlations were computed between wall thickness and plaque-thickness/area at 1 and 4.5 years separately within the stented grafts group; time points were not combined for this analysis due to the same subjects providing data in each, while Pearson assumes between-subject independence.

## Results

### Patient characteristics

Of the 30 patients who underwent randomization and treatment with the VEST in the original trial, MACCE data were available for all patients, and angiography and IVUS measurements were available for 21 patients (70%) at a mean (±SD) 4.5 ± 0.3 year follow-up. Of the 9 patients that did not return for angiographic follow-up, 2 had both vein grafts previously known to be occluded and were not asked to return, 3 were deceased, and 4 refused the procedure. Demographic and medical history data are provided in Table 1.

**Table 1:** Clinical characteristics of all patients at randomization and those who underwent follow-up angiography

Characteristic	All patients (*n* = 30)	Angiographic follow-up (*n* = 21)
Age (years)	65 ± 8	65 ± 9
Male	27 (90)	16 (89)
Smoking status:		
Current	3 (10)	2 (10)
Ex-smoker	22 (73.3)	15 (71)
Never	5 (16.7)	4 (19)
Diabetes:		
IDDM	5 (17)	3 (14)
NIDDM	6 (20)	5 (24)
No history	19 (63)	13 (62)
Hypertension:	20 (66.7)	14 (67)
Hyperlipidemia	29 (96.7)	20 (95)
Prior stroke (non-debilitating)	1 (3.3)	1 (5)
COPD	1 (3.3)	0
NYHA class:		
I	11 (36.7)	6 (29)
II	13 (43.3)	9 (43)
III	4 (13.3)	4 (19)
IV	2 (6.7)	2 (9)
LVEF (%)	56 ± 10	55 ± 10
Creatinine (umol/L)	85 ± 18.9	81.7 ± 18.1
Pre-op Logistic EuroSCORE (%)	1.58 ± 1.29	1.7 ± 1.4
Total number grafts/patient	3.3 ± 0.5	3.4 ± 0.5
Days of follow up	1601 ± 102	1601 ± 113

Mean (±SD) or n (%). *IDDM* insulin dependent diabetes, *NIDDM* non-insulin dependent diabetes, *COPD* chronic obstructive pulmonary disease, *NYHA* New York Heart Association, *LVEF* left ventricular ejection fraction

### Clinical outcomes

Three patients (10%) had died since surgery; one was a cardiac-related death and two were due to cancer. Overall 3 patients (10%) experienced one or more MACCE events. Of 3 revascularization events, one was triggered by the protocol-mandated angiographic control, one was to the nonstented territory, and one was to both stented and nonstented territories. One myocardial infarction (3.3%) and no strokes were reported.

### Vein graft disease markers

**Fig. 1:**
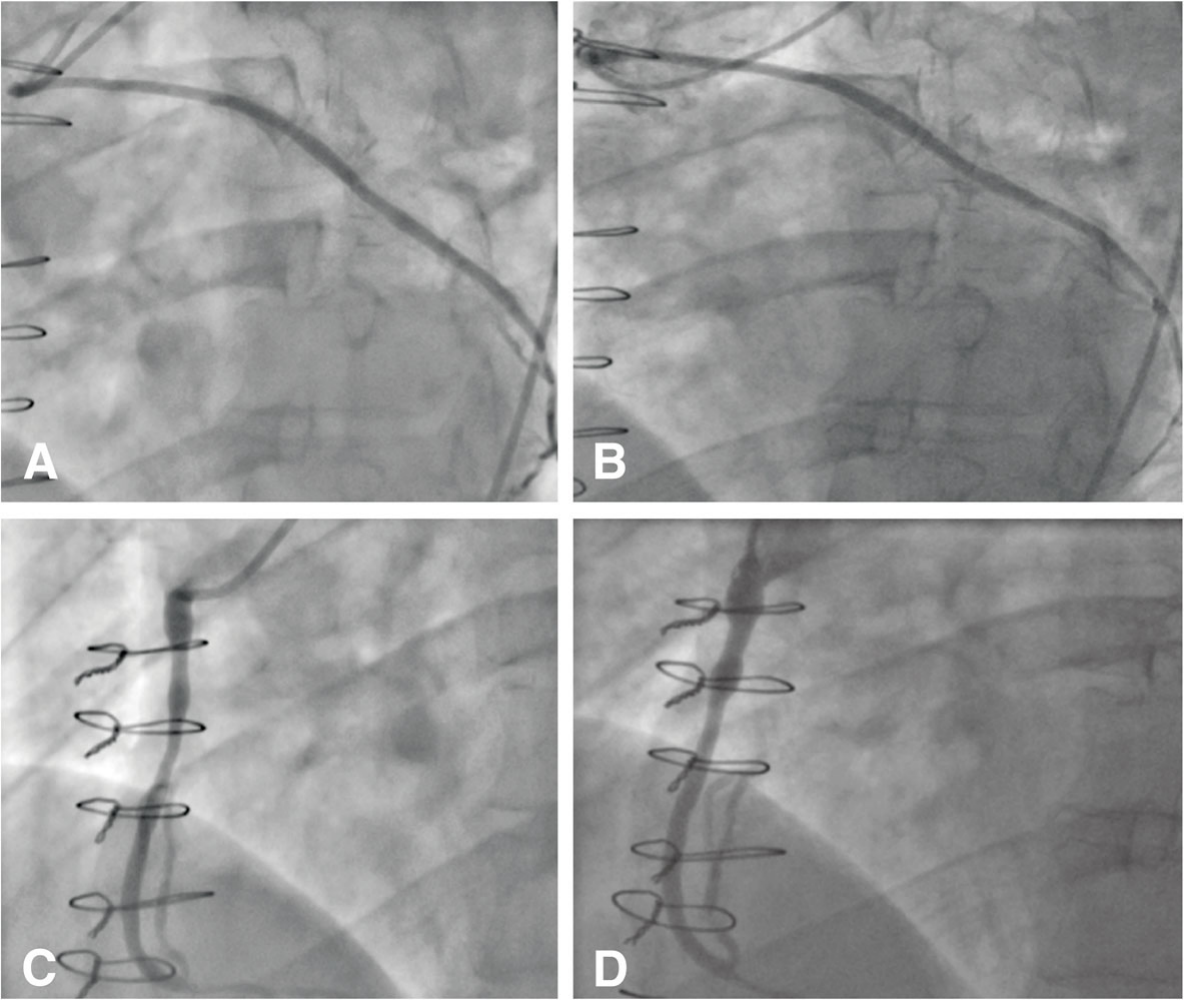
Angiographic images showing a within-patient comparison of stented and nonstented SVGs at 1 and 4.5 year follow-up. Stented SVG to obtuse marginal artery at 1 year (**a**) and after 4.5 years (**b**). Nonstented SVG to right coronary artery at 1 year (**c**) and 4.5 years (**d**)

**Fig. 2:**
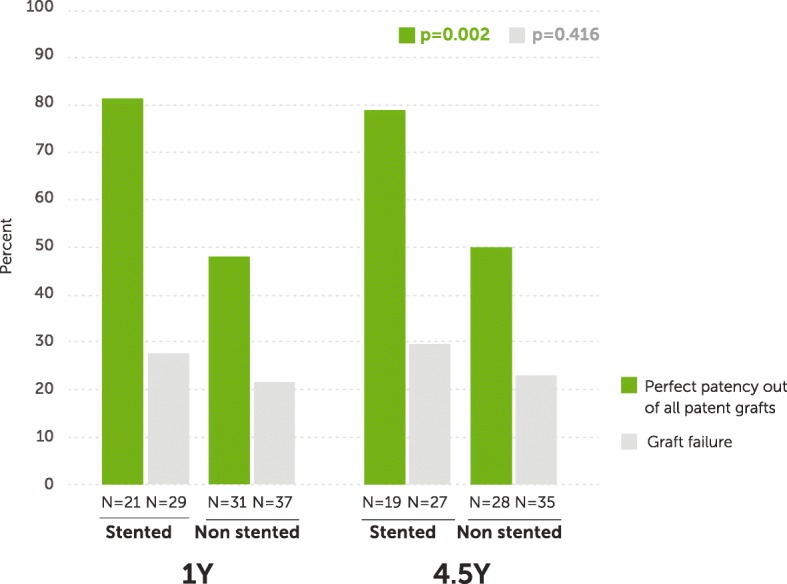
Perfect patency and graft failure rates of stented and nonstented vein grafts at 1 and 4.5 years post CABG. Green bars - perfect patency rates within each arm of stented (*n* = 40) and nonstented (*n* = 59) grafts (*p* = 0.002). Grey bars - graft failure rates within each arm of stented (*n* = 56) and nonstented (*n* = 72) grafts (*p* = 0.416)

Figure 1 shows angiographic images of a within-patient comparison of stented and nonstented SVGs at 1 and 4.5 year follow-up. Vein graft failure rates at 4.5 years were comparable between the stented and non-stented graft groups (Fig. 2). In the stented group, all grafts that were patent at the 1 year follow up, maintained their patency at the 4.5 year follow up, however in the non-stented group there was one additional graft occlusion between 1 and 4.5 years. At 4.5 years, stented vein grafts maintained perfectly-patent lumens without irregularities at significantly higher proportions than non-stented grafts (Fig. 2).

**Fig. 3:**
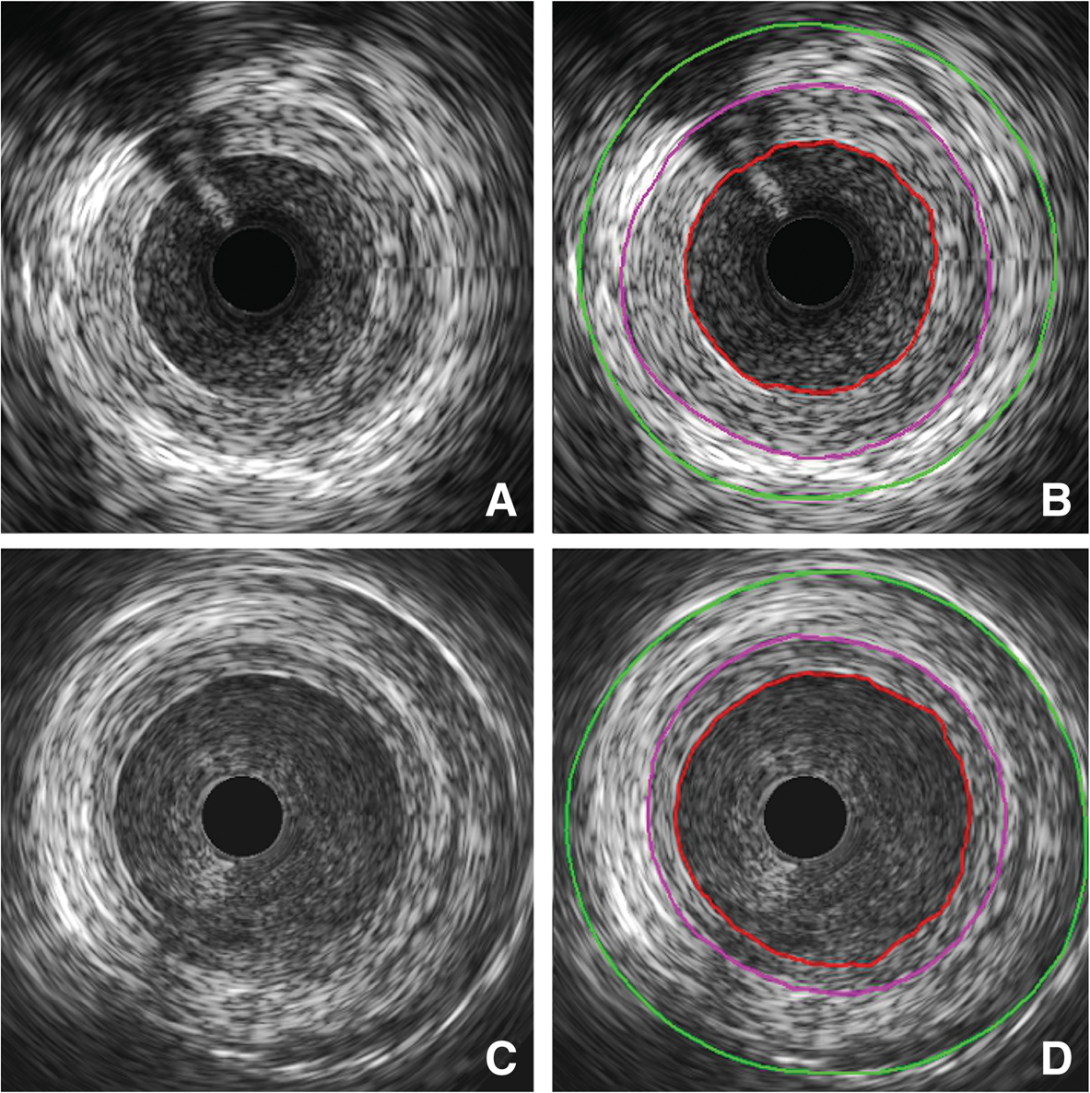
Within-patient comparison of intimal hyperplasia using IVUS. Segment of a nonstented SVG to the first obtuse marginal 4.5 years after implantation without (**a**) and with (**b**) marking of the lumen (red), EEM (purple) and outer vessel (green). Segment of externally stented SVG to the second obtuse marginal 4.5 years after implantation without (**c**) and with (**d**) marking of the lumen (red), EEM (purple) and stent (green)

**Fig. 4:**
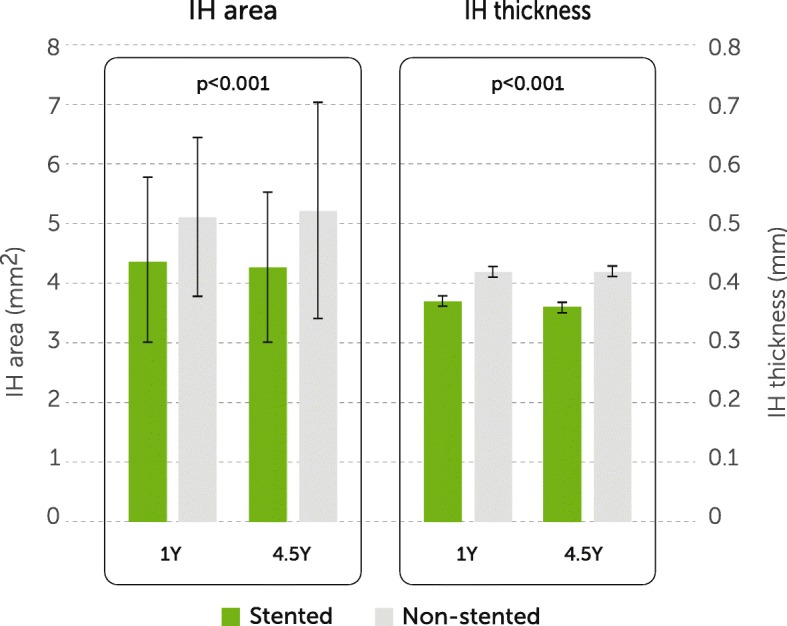
Comparison of intimal hyperplasia proliferation markers at 1 and 4.5 year follow-up. Data are mean ± SD. IH, intimal hyperplasia; 1Y, 1 year follow-up; 4.5Y, 4.5 year follow-up. Green square Stented grafts, grey square Non-stented grafts

Fig. 3 illustrates IVUS images of a within-patient comparison of stented and nonstented SVGs at 1 and 4.5 year follow-up. IH area and thickness at 4.5 years did not change significantly compared to 1 year but both were significantly reduced in the stented versus the nonstented grafts (Fig. 4). Lumen diameters did not significantly differ between the two groups (3.38 ± 0.50 mm vs. 3.45 ± 0.54 mm stented vs. nonstented; *p* = 0.585).

**Fig. 5:**
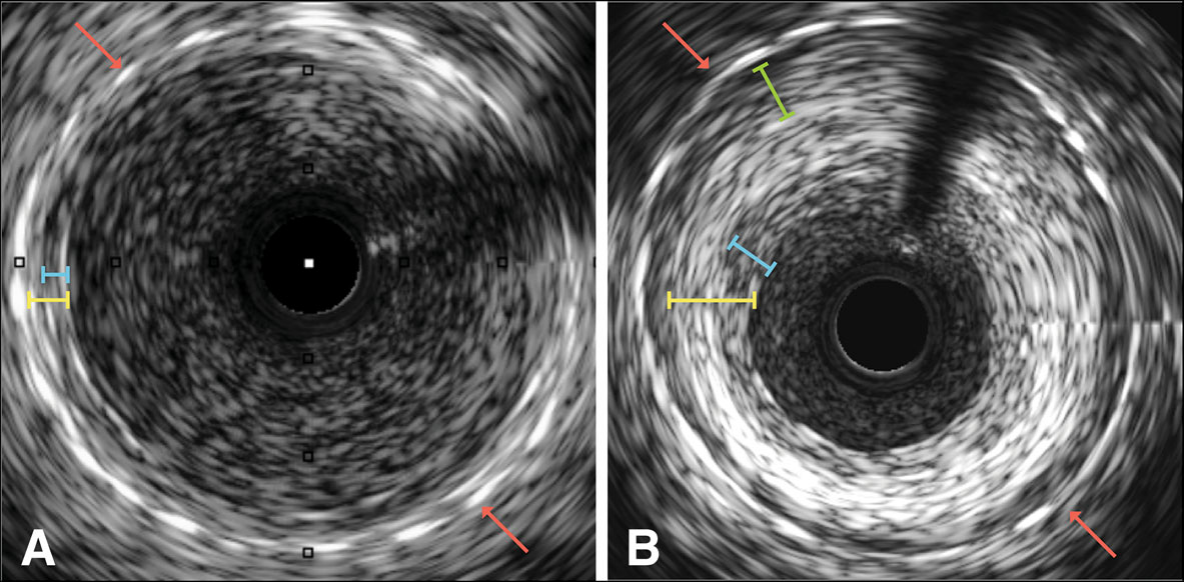
IVUS images showing **a** constrictive versus **b** loose fitting external stent (red arrows). Difference in wall thickness (yellow) and intima layer (blue) can be observed. In addition, there is formation of “neo-adventitia” between the loose-fitting stent and the original vessel wall

**Fig. 6:**
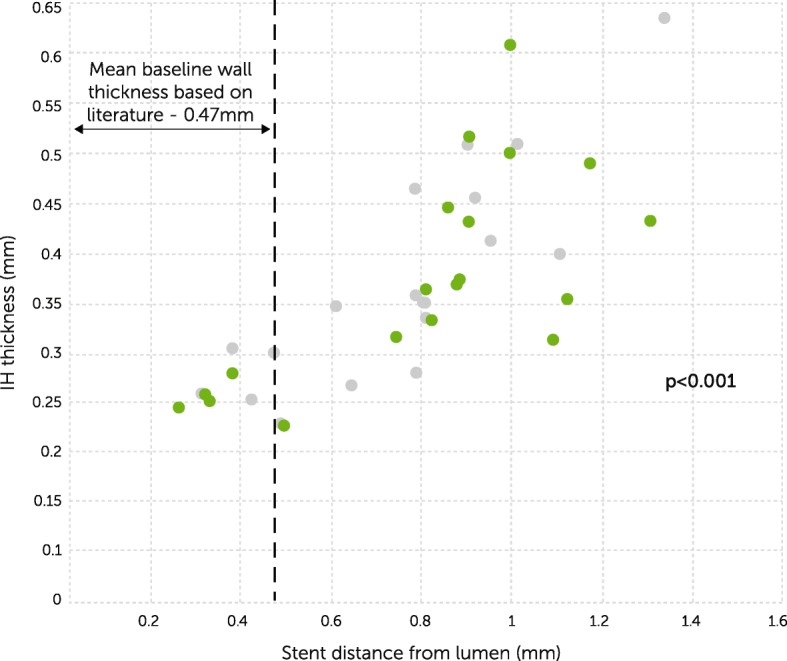
Intimal hyperplasia thickness correlated with distance of the external stent from the lumen. grey circle 1 year (correlation coefficient 0.76, *p* < 0.001), green circle 4.5 years (correlation coefficient 0.84, *p* < 0.001). Baseline saphenous vein wall thickness (−--) is derived from relevant literature [30, 31]

Grafts with Fitzgibbon I classification had significantly lower plaque area compared to grafts with Fitzgibbon II/III (*p* = 0.04, Table 2). Figure 5 illustrates IVUS images of a constrictive- and a loose-fitting external stent. The distance of the stent to SVG wall was positively correlated with IH thickness both at 1 year (correlation coefficient 0.76, *p* < 0.001) and at 4.5 years (correlation coefficient 0.84, *p* < 0.001; Fig. 6).

**Table 2:** Cross sectional IVUS parameters for Fitzgibbon grade vein graft groups

Variable	Fitzgibbon I	Fitzgibbon II & III	*P* value
IH area [mm^2^]	4.5 (3.9, 5.0)	5.2 (4.5, 5.8)	0.04
IH thickness [mm]	0.4 (0.3, 0.4)	0.4 (0.4, 0.5)	0.09
Lumen diameter [mm]	3.4 (3.2, 3.6)	3.5 (3.3, 3.8)	0.38

Data are mean (95% CI). *IH* intimal hyperplasia

## Discussion

VEST IV is a unique study, presenting for the first time the long-term follow-up of a randomized controlled trial evaluating the impact of external stenting of SVGs used in CABG surgery. Our key finding is that the benefits of external stents on improving Fitzgibbon perfect patency and reducing IH that was demonstrated at 1 year are maintained at 4.5 years. Similarly, with the exception of an additional vein graft occlusion in the nonstented group, there was little further deterioration at longer-term follow up. A second key finding, as discussed subsequently, is that the distance of the VEST stent from the SVG lumen was an important determinant of the degree of IH. The MACCE rate of 18% in this study was comparable to that reported by the SYNTAX trial group for their 3-vessel disease subgroup at 4 years and at 5 years post CABG at 19 and 24% respectively [11, 12].

This study strengthens and corroborates previous pre-clinical and clinical reports suggesting a protective biomechanical effect of a braided cobalt chrome external stent on vein graft remodeling [7, 8, 13, 14]. A particular strength of our trial was the paired study design with each patient acting as their own control, thereby eliminating many of the potential factors that could affect SVG disease progression. The stented and nonstented graft groups were well balanced with respect to baseline anatomical and physiological parameters that might contribute to the development of IH, including the diameter of the native coronary artery and the severity of the proximal coronary artery stenosis. This was also evidenced by the similarity of measured graft flows in both the stented and nonstented SVGs, intraoperatively by TTFM and at 1-year by angiography [7]. Furthermore, while previous IVUS studies on SVGs were limited only to the proximal part of the grafts at one time point [15], our study provides, for the first time, new insight into the diffuse nature of the disease by recording intimal development along the entire SVG length at both 1 and 4.5 years.

The early phase of vein graft remodeling is dominated by luminal enlargement followed by a later phase of vein graft thickening and IH. Luminal enlargement is generated mainly by the exposure to the high shear stress of the arterial circulation [3]. During the first months post implantation, as a reaction to the elevated wall tension, there is a proliferative thickening of the venous wall and changes in wall composition [3]. Hozumi et al. have shown that between 1 to 12 months post implantation, intimal area was significantly increased from 0.90 mm^2^ to 5.26 mm^2^ (*p* < 0.001) [15]. Our findings concur with Hozumi et al., suggesting that most SVG remodeling occurs over the first year after grafting and, consequently, that early intervention is crucial in order to effectively lessen the pathological changes which serve as the foundation for occlusive atheromatous lesions 5–10 years following surgery [15]. The mitigation of IH and lumen deformation achieved by external stenting was most pronounced in the first year after implantation and was maintained over the subsequent 3.5 years. This observation is also in line with experimental data on biodegradable external stents which demonstrated that the majority of the inhibitory effect on SVG remodeling was achieved 6 months post implantation [16].

In both arteries and veins, lumen irregularities generate low and oscillatory shear stress that is directly correlated with accelerated vascular disease [13, 17]. A high proportion of SVGs, mainly from the upper leg, demonstrate caliber irregularities even during harvesting while > 25% of SVG show severe segmental ectasia with > 50% dilatation at 1 year [7]. In their landmark publication, Fitzgibbon and colleagues described the progression of SVG disease over the course of 15 years [9]. At 5, 10 and 15 years, only 52, 23 and 19% of the patent grafts respectively demonstrated “perfect” patency with no lumen irregularities. The diffuse progressive nature of vein graft disease was further supported by contemporary studies that demonstrate that only 12–25% of SVG are perfectly patent 10 years after surgery [18]. We found that reduced SVG lumen irregularity is associated with decreased intimal area and thickness, most probably due to the improved hemodynamics and reductions in oscillatory shear stress provided by external stents [13, 17]. External stenting prevented SVG deformation early after implantation and mitigated development of further lumen irregularities at 4.5 years.

Mechanical external support of SVGs has been a focus of intense research, with their use intended to reduce well-documented pathophysiological changes that occur in the SVG following implantation. These devices have developed substantially over recent years, and there is now a large body of data in both animal models and human patients related to their biomechanical effects [17, 19, 20]. External stenting targets some of the key factors initiating the pathological cascade in SVG post implantation such as high circumferential wall stress and disturbed flow patterns due to luminal irregularities [13, 14, 17]. The rationale and the physiological basis for mechanical external stents is further supported by the reported benefits of the “no touch” technique, in which the saphenous vein is harvested with a pedicle of surrounding tissue that serves as an external support with both mechanical and biological roles [21]. Randomized prospective studies showed that no touch technique is associated with superior patency rates compared with conventionally prepared vein grafts both in the short-term (18 months) and long-term (8.5 years) at angiographic follow-up [22, 23].

Early clinical experience with external stents showed conflicting data with early patency rates ranging from 28 to 92% at 6–12 months [24, 25]. High SVG failure rates were attributed to the design of the first generation of external stents that required incorporation of the device into the anastomoses, constriction of the graft and fixation of the stent to the SVG with fibrin glue [25]. Several studies have shown that fibrin glue on the external surface of vein grafts lead to aneurysmal degeneration and excessive IH which may jeopardize vein graft patency [25–27]. Second generation technologies which did not require the use of fibrin glue or incorporation to the anastomoses site showed comparable patency rates to non-stented SVG at 1 year [7].

The appropriate size of external stents for vein grafts has been a focus of controversy and another possible confounding factor for success of external stenting. Zilla and colleagues concluded that constrictive external stents, which reduce diameter mismatch with the target artery, are more effective in mitigating IH compared to non-restrictive stents [28]. In contrast, Izzat and colleagues have shown that loose-fitting external stents are more effective in suppressing intimal proliferation [29]. The rationale behind using oversized external supports was to provide sufficient space to promote adventitial neovascularization, which was potentially interrupted by constrictive stents [20]. The distance of the external stent from the lumen is equivalent to the SVG wall thickness. The closer this measure is to the vein baseline wall thickness, the better the conformity of the VEST to the vein. We postulated that a closer conformity would improve VEST performance in mitigating IH, and indeed we found that the correlation between the distance of the VEST from the lumen and the IH thickness was indeed significant in our study. As shown in Fig. 6, the closer the value is to the baseline literature reported vein wall thickness, the lesser the proliferation of IH [30, 31]. This finding suggests that mildly constricting the vein has a beneficial effect on intimal proliferation, most probably due to more effective reduction in wall tension, SVG dilatation, and lumen irregularities. It is expected that future studies, in which appropriate model selection will ensure a mildly constrictive stent, a more effective reduction in IH will be achieved.

### Limitations

Together with the small sample size, a further limitation of our study is that the study cohort was based on a first in human trial and exhibited learning curve effects of both the technology and the implantation technique (which were largely resolved in VEST II [8]). The technical failures and inappropriate model selection, with suboptimal dimensional match between the SVG and the stent, likely have adversely affected the stent’s ability to even more effectively mitigate IH. VEST III, a large randomized trial that is currently underway (NCT02511834), will address both the population size and learning curve issues.

## Conclusion

Improving SVG longevity may have a direct impact on the clinical outcome of CABG and patients’ quality of life. Our study provides novel insights into the biomechanical effects of external stenting of SVGs and their long-term performance – to our knowledge, the only long-term follow-up of external stenting to SVGs to date. If the beneficial effect of external stenting that we observed at one and 4.5 years is maintained over a 10–15 year period, it may have a substantial impact on improving clinical outcomes. Future and ongoing large randomized trials with long-term follow up will further determine the role of external stents in CABG surgery.

## Abbreviations

CABG: Coronary artery bypass graft
EEM: External elastic lamina
IH: Intimal hyperplasia
IVUS: Intravascular ultrasound
MACCE: Major cardiovascular and cerebral events
MI: Myocardial infarction
SD: Standard deviation
SVG: Saphenous vein graft
